# The Myth of Myocardial Infarction With Normal Coronary Angiography

**DOI:** 10.7759/cureus.13662

**Published:** 2021-03-03

**Authors:** Ziad A Taher, Abdulhalim J Kinsara

**Affiliations:** 1 Department of Medicine, King Abdullah International Medical Research Center, Jeddah, SAU; 2 Department of Medicine, Ministry of National Guard-Health Affairs, Jeddah, SAU; 3 Department of Cardiology, Ministry of National Guard-Health Affairs, King Saud Bin Abdulaziz University for Health Sciences, Com-Wr. King Abdullah International Medical Research Center, Jeddah, SAU

**Keywords:** myocardial infarction of no obstructive atherosclerosis, pathophysiology, acute coronary syndrome

## Abstract

Myocardial infarction with no obstructive atherosclerosis is an increasingly recognized presentation of acute coronary syndrome (ACS). The disease has all the clinical features of an ACS, but the only exception is that the coronary angiogram indicates non-obstructive coronary artery disease. Although different pathophysiological mechanisms have been postulated, no definitive mechanism has been identified. Consequently, the treatment plan varies and depends on the more probable mechanism. Here, we review the current body of knowledge about this disease and discuss updated management strategies.

## Introduction and background

Myocardial infarction with no obstructive atherosclerosis (MINOCA) is defined clinically as the presence of myocardial infarction (MI) features (i.e., symptoms, electrocardiogram [ECG], laboratory, and imaging) in the absence of obstructive coronary artery disease, a <50% stenosis without a clear cause for presentation. It was first described by Gross and Sternberg in 1939 [1]. Various terms have been used for the condition, including MINOCA, myocardial infarction with normal coronary arteries, and ischemia and no obstructive coronary artery disease or troponin-positive non-obstructive coronary arteries [2]. MINOCA has been incorporated in the Fourth Universal Definition of acute MI.

MINOCA has a variable prevalence, ranging from 1% to 25% [3-5]. A recent study indicated a prevalence of 8.8% of all MI patients [6]. Women are affected five times more than males; however, in China, males are predominantly affected.

## Review

Etiology and diagnosis

MINOCA is credited with some known and unknown causes, resulting in a classification of epicardial and microvascular causes.

Epicardial Causes

Cornary plaque disruption is an example of an epicardial cause. According to literature, 20%-40% of MINOCA cases are secondary to coronary plaque disruption [7]. The disruption cannot be visualized by coronary angiography if it grew extraluminally or expanded outward. A preferable method of detection is an intravascular ultrasound (IVUS) as both the layers of the vessel and the lesion can be seen. The prognosis of patients with coronary plaque disruption depends largely on plaque remodeling and a cardiovascular event such as acute coronary syndrome (ACS) [8].

Coronary Artery Dissection

Although it can cause luminal obstruction, it may not be apparent on angiography, facilitating the diagnosis of MINOCA [9]. The etiology is still vague, but some pathological features are noticed in the diseased artery, such as fibromuscular dysplasia [10]. Young women are more likely to have coronary artery dissection based on literature proposing that hormones, pregnancy, and delivery increase the risk [11]. The highest chance of a positive diagnosis and evaluation of MINOCA is by IVUS in the context of a high recurrence rate [11].

Coronary Artery Spasm

Coronary artery spasm (CAS) is an uncontrolled smooth muscle contraction observed in some obstructive MI cases. It can be provoked by other triggers, resulting in uneventful MINOCA. Usually, it occurs in a specific section of the epicardial arteries; however, it may also involve more than one section of an artery or even more than one artery [12]. CAS constitutes 27% of all MINOCA cases, as reported in some studies; however, other studies report a rate of up to 95%, depending on the difference of the definition of spasm used in each study, ethnicity, and the trigger substance [13,14]. A patient with MINOCA is expected to experience CAS with recurrent episodes of angina at rest, which responds to short-acting nitrates, principally when there are transient ischemic ECG changes.

Coronary Thromboembolism/Embolism

This condition can also involve the microcirculation of the coronary arteries, resulting in MINOCA. The factors contributing to this mechanism include thrombotic disorders and arterial thrombi. MINOCA, secondary to hereditary thrombotic disorders, constitutes 14% of the cases [2].

Myocarditis

Myocarditis is responsible for 33% of all MINOCA cases [15]. The primary investigation when myocarditis is suspected includes cardiovascular magnetic resonance imaging (CMR); however, a biopsy is the only way to identify the cause and to confirm the diagnosis.

Takotsubo Syndrome

This syndrome is unique because it presents as acute heart failure (HF) in patients with MINOCA [16]. They present with features of HF in an echocardiography such as dyskinesia of the LV-mid segment and regional wall motion abnormality, with ST-segment elevation in the ECG. However, it is difficult to diagnose because the troponin level is not high in Takotsubo syndrome.

MINOCA With Uncertain Etiology

CMR is preferred as it can reveal the cause in many cases. However, in some cases, CMR result would be normal, for example, vasospastic angina and coronary plaque disruption. In these cases, an IVUS is preferable [17]. Non-cardiac disorders that present as MINOCA include, but are not limited to, stroke, pulmonary embolism, sepsis, adult respiratory distress syndrome, and end-stage renal failure.

Diagnosis

**Figure 1:**
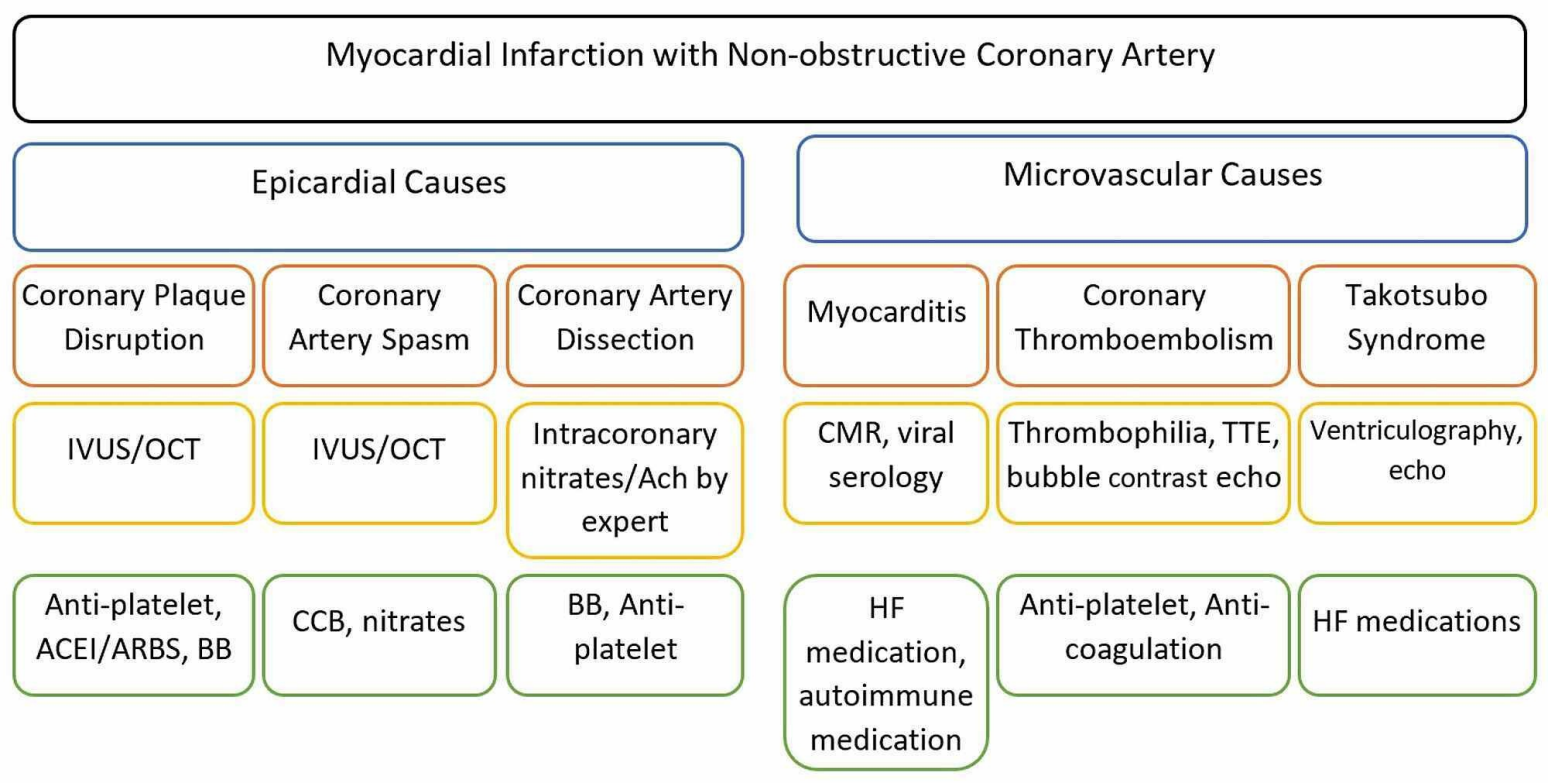
Classification, diagnosis, and management of myocardial infarction with non-obstructive coronary artery. IVUS: intravenous ultrasound; OCT: optical coherence tomography, Ach: acetylcholine, CMR: cardiac magnetic resonance, TTE: transthoracic echocardiography; ACEI: angiotensin-converting enzyme inhibitor, ARBS: angiotensin receptor blocker; BB: beta-blocker; CCB: calcium channel blocker; HF: heart failure

Coronary angiography is routinely done based on the acute presentation and when more diagnostic information is required. Echocardiography, in particular, is valuable to rule out other differential diagnoses. CMR detects myocardial fibrosis or scarring, but it has to be delayed for at least two weeks after the acute onset (Figure 1). A study indicated a significant number with ischemic late gadolinium enhancement changes or myocardial wall edema. Patients with CMR abnormalities also had a higher level of troponin [18].

 

Management of myocardial infarction with no obstructive atherosclerosis

The management of MINOCA depends on the underlying etiology. Plaque disruption is managed with dual antiplatelet treatment for 12 months, followed by single life-long antiplatelet therapy. Statin therapy is also suggested for any degree of atherosclerosis [19].

CAS is best managed with vasodilators, such as nitrates and calcium channel blockers. Refractory CAS is observed in 10%-20% of all cases. In this scenario, fasudil was shown to be effective in a Japanese population. In a specific patient, a stent implantation and sympathetic denervation is acceptable. An implantable cardiac defibrillator is limited to high-risk patients [12].

For coronary artery dissection, no clinical trials have been performed to date to indicate a definitive management strategy. Conservative management is preferred in some cases. In fact, some interventions like stenting could worsen the patient’s situation [20].

Microvascular causes such as myocarditis are managed with supportive therapy targeting the underlying cause. Antiviral therapy or immunosuppression therapy should be considered when the underlying cause is identified. Empiric therapeutic approaches are recommended in coronary thromboembolism. These include emergency coronary artery bypass grafting, stent implantation, and intra-coronary thrombolysis.

Takotsubo syndrome recovers spontaneously weeks after MINOCA. The complications of the syndrome are similar to MI. Additionally, symptoms of HF may persist for a long time, secondary to impaired metabolic indices, fibrosis, and deformation [21].

The hospital mortality rate of MINOCA varies from 0% to 8%. The one-year mortality rate is 5.6%. A small proportion (9.9%) of patients are prone to develop a major cardiovascular or cerebrovascular event per year [22,23]. The prognosis of myocarditis is excellent; in 50% of the cases, it resolves over two to four weeks [24]. However, 12%-25% may deteriorate to HF requiring a transplant.

## Conclusions

MINOCA is a unique form of acute coronary artery syndrome. The suggested etiologies include coronary plaque disruption, CAS, coronary artery dissection, coronary thromboembolism, myocarditis, and Takotsubo syndrome. Management options aim to reduce the recurrence of MI by controlling the underlying risk factors. More research is warranted to optimize better management options.

## References

[1] Gross H, Sternberg H (1939). Myocardial infarction without significant lesions of coronary arteries. Arch Intern Med.

[2] Pasupathy S, Air T, Dreyer P, Tavella R, Beltrame JF (2015). Systematic review of patients presenting with suspected myocardial infarction and nonobstructive coronary arteries. Circulation.

[3] Gehrie ER, Reynolds HR, Chen AY (2009). Characterization and outcomes of women and men with non-ST-segment elevation myocardial infarction and nonobstructive coronary artery disease: results from the can rapid risk stratification of unstable angina patients suppress adverse outcomes with early implementation of the ACC/AHA guidelines (CRUSADE) quality improvement initiative. Am Heart J.

[4] Bugiardini R, Manfrini O, De Ferrari M (2006). Unanswered questions for management of acute coronary syndrome: risk stratification of patients with minimal disease or normal findings on coronary angiography. Arch Intern Med.

[5] Kang WY, Jeong MH, Ahn YK (2011). Are patients with angiographically near-normal coronary arteries who present as acute myocardial infarction actually safe?. Int J Cardiol.

[6] Planer D, Mehran R, Ohman EM, White HD, Newman JD, Xu K, Stone GW (2014). Prognosis of patients with non-ST-segment-elevation myocardial infarction and nonobstructive coronary artery disease: propensity-matched analysis from the Acute Catheterization and Urgent Intervention Triage Strategy trial. Circ Cardiovasc Interv.

[7] Ouldzein H, Elbaz M, Roncalli J, Cagnac R, Carrié D, Puel J, Alibelli-Chemarin MJ (2012). Plaque rupture and morphological characteristics of the culprit lesion in acute coronary syndromes without significant angiographic lesion: analysis by intravascular ultrasound. Ann Cardiol Angeiol.

[8] Rossini R, Capodanno D, Lettieri C (2013). Long-term outcomes of patients with acute coronary syndrome and nonobstructive coronary artery disease. Am J Cardiol.

[9] Alfonso F, Paulo M, Dutary J (2012). Endovascular imaging of angiographically invisible spontaneous coronary artery dissection. JACC Cardiovasc Interv.

[10] Saw J, Aymong E, Sedlak T (2014). Spontaneous coronary artery dissection: association with predisposing arteriopathies and precipitating stressors and cardiovascular outcomes. Circ Cardiovasc Interv.

[11] Scalone G, Niccoli G, Crea F (2019). Editor's choice- pathophysiology, diagnosis and management of MINOCA: an update. Eur Heart J Acute Cardiovasc Care.

[12] Lanza GA, Sestito A, Sgueglia GA, Infusino F, Manolfi M, Crea F, Maseri A (2007). Current clinical features, diagnostic assessment and prognostic determinants of patients with variant angina. Int J Cardiol.

[13] Kaski JC, Crea F, Meran D (1986). Local coronary supersensitivity to diverse vasoconstrictive stimuli in patients with variant angina. Circulation.

[14] Pristipino C, Beltrame JF, Finocchiaro ML (2000). Major racial differences in coronary constrictor response between Japanese and Caucasians with recent myocardial infarction. Circulation.

[15] Tornvall P, Gerbaud E, Behaghel A (2015). Myocarditis or "true" infarction by cardiac magnetic resonance in patients with a clinical diagnosis of myocardial infarction without obstructive coronary disease: a meta-analysis of individual patient data. Atherosclerosis.

[16] Prasad A (2007). Apical ballooning syndrome: an important differential diagnosis of acute myocardial infarction. Circulation.

[17] Reynolds HR, Srichai MB, Iqbal SN (2011). Mechanisms of myocardial infarction in women without angiographically obstructive coronary artery disease. Circulation.

[18] Tayal B, Freeman P, Ericsson F (2020). Characterisation of patients with and without cardiac magnetic resonance imaging abnormalities presenting with myocardial infarction with non-obstructive coronary arteries (MINOCA). Acta Cardiol.

[19] Takarada S, Imanishi T, Ishibashi K (2010). The effect of lipid and inflammatory profiles on the morphological changes of lipid-rich plaques in patients with non-ST-segment elevated acute coronary syndrome: follow-up study by optical coherence tomography and intravascular ultrasound. JACC Cardiovasc Interv.

[20] Tweet MS, Hayes SN, Pitta SR (2012). Clinical features, management, and prognosis of spontaneous coronary artery dissection. Circulation.

[21] Templin C, Ghadri JR, Diekmann J (2015). The clinical features and outcomes of Takotsubo (stress) cardiomyopathy. N Engl J Med.

[22] Glaveckaitė S, Šerpytis P, Pečiūraitė D, Puronaitė R, Valevičienė N (2016). Clinical features and three-year outcomes of Takotsubo (stress) cardiomyopathy: observational data from one center. Hellenic J Cardiol.

[23] Elesber AA, Prasad A, Lennon RJ, Scott Wright R, Lerman A, Rihal CS (2007). Four-year recurrence rate and prognosis of the apical ballooning syndrome. J Am Coll Cardiol.

[24] Sliwa K, Hilfiker-Kleiner D, Petrie MC (2010). Current state of knowledge on aetiology, diagnosis, management, and therapy of peripartum cardiomyopathy: a position statement from the Heart Failure Association of the European Society of Cardiology Working Group on peripartum cardiomyopathy. Eur J Heart Fail.

